# Lack of pathogen identification influencing antibiotic de-escalation in hospital-acquired pneumonia

**DOI:** 10.1017/ash.2022.325

**Published:** 2022-11-07

**Authors:** Matthew C. Bittmann, Scott T. Micek, Marin H. Kollef

**Affiliations:** 1 Division of Medical Education, Washington University School of Medicine, St. Louis, Missouri; 2 Department of Pharmacy Practice, University of Health Sciences and Pharmacy, St. Louis, Missouri; 3 Division of Pulmonary and Critical Care Medicine, Washington University School of Medicine, St. Louis, Missouri

Hospital-acquired pneumonia (HAP) is the most common hospital-acquired infection, representing ∼22% of all hospital-acquired infections.^1^ Also, antibiotic resistance is a growing concern throughout healthcare.^2,3^ Studies on de-escalation of antibiotic therapy in HAP have shown outcomes to be no worse for patients who had antibiotics de-escalated compared with those who did not.^4–6^ Therefore, it is prudent to further characterize existing rates of antibiotic de-escalation among HAP patients. In this study, we sought to determine whether any systematic differences in de-escalation patterns exist between 4 pathogen groups (ie, Enterobacterales, *Staphylococcus aureus, Pseudomonas aeruginosa*, and bacteria-culture–negative status) in patients with HAP and ventilator-acquired pneumonia (VAP) pneumonia.

## Methods

The methods used to create this database have been described previously.^4^ This retrospective cohort comprised patients was admitted to Barnes Jewish Hospital, a large academic center in St. Louis, Missouri, between January 16, 2016, and December 31, 2019. The study was approved by the Washington University institutional review board. Adult patients with ICD-9 or ICD-10 codes and who met the following criteria for HAP or VAP were included: (1) index date of infection onset ≥48 hours after hospital admission for HAP or ≥48 hours after initiation of mechanical ventilation for VAP, (2) chest radiograph completed within ±24 hours of index pneumonia date, (3) active orders for either anti–methicillin-resistant *Staphylococcus aureus* (MRSA) or anti–*Pseudomonas aeruginosa* antibiotics, and (4) at least 1 sign of infection, including a white blood cell count ≥11 or ≤4×10^9^ cells/L or temperature ≥38 or ≤36°C. The index date of pneumonia diagnosis was defined as day 0. Pharmacy-verified intravenous and oral antibiotic orders of interest were collected for each day of admission.

Antibiotic spectrum scores modified based on the Barnes-Jewish Hospital antibiogram and the addition of antibiotics not listed in the original description reported by Gerber et al^7^ were calculated on day 0 and for each day thereafter.^4,7^ Microbiologic data, including respiratory cultures, respiratory viral polymerase chain reaction tests, MRSA nasal culture swabs, and blood cultures, were collected from days –1 to 3 of index pneumonia diagnosis. Respiratory cultures were classified as sputum-like specimens, which included sputum and tracheal aspirates, and bronchoscopy specimens, which included bronchoalveolar lavage, washings, and brushings. Respiratory samples identified as positive for a likely pathogen excluded those with only yeast, fungal structural elements, and/or clinically insignificant flora. Among the blood cultures identified as positive for a likely pathogen, we excluded those with only coagulase-negative staphylococci. Culture negative was defined as no growth from any of the cultures described above.

Study participants were categorized in 4 groups based on the pathogen associated with their pneumonia: *Staphylococcus aureus* (n = 134), *Pseudomonas aeruginosa* (n = 59), *Enterobacterales* spp (n = 87), and bacteria-culture negative (n = 1,404). Due to small sample sizes, study participants with pathogens not of interest were excluded from analysis. These pathogens included *Stenotrophomonas*, *Burkholderia*, and *Acinetobacter* as well as *S. pneumoniae*, *H. influenzae*, and *S. agalactiae*.

The spectrum scores from each pathogen group were compared separately on days 0, 3, 7, 10, and 14 using Kruskal-Wallace tests and subsequent Dunn post-hoc tests if warranted. The initial spectrum scores were calculated on the day study participants had a new active order for either anti-MRSA or anti–*Pseudomonas aeruginosa* antibiotics and at meeting the study criteria for HAP and VAP described above. The composite outcome of all-cause mortality and hospital readmission within 30 days of pneumonia diagnosis was also calculated similar to our prior study.^4^ All analyses were performed using R version 4.1.3 and R Studio version 2022.02.3 (R Foundation for Statistical Computing, Vienna, Austria).

## Results

The demographic characteristics of this cohort have been described previously.^4^ In total, 11,860 admissions were screened for inclusion, and 1,684 of these met inclusion criteria for this study. The final cohort comprised 87 patients (5.2%) with *Enterobacterales* infections, 134 (7.9%) with *Staphylococcus aureus* infections, 59 (3.5%) with *Pseudomonas aeruginosa* infections. In addition, 1,404 (83.4%) had bacteria-culture–negative pneumonia. The median age was 62 years (interquartile range [IQR], 51–70.8). In total, 975 study participants (57.9%) were male, and 1,231 (73.1%) were white. The median Charlson comorbidity index score was 6 (IQR, 3.8–8). Overall, 1,057 patients (62.8%) were diagnosed with HAP and 627 patients (37.2%) were diagnosed with VAP. 1483 (88.1%) patients were in the ICU on the day of diagnosis.

**Fig. 1.**
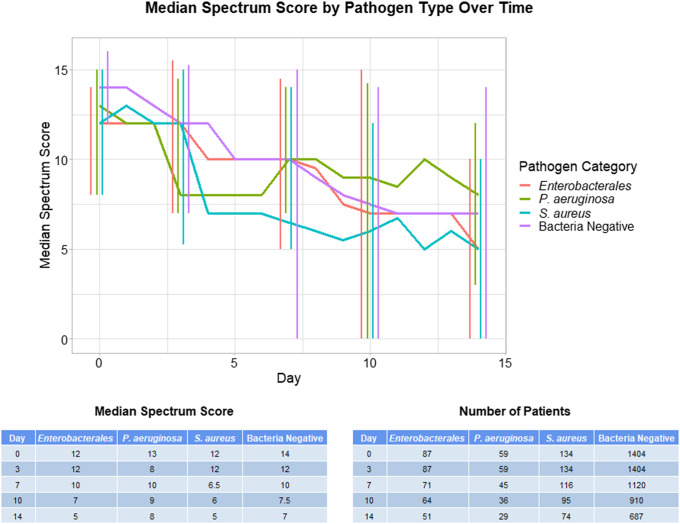
Median antibiotic spectrum scores over time on days 0, 3, 7, 10, and 14. Error bars indicate interquartile range.

In comparing spectrum scores between pathogen groups on days 0, 3, 7, 10, and 14, only day 0 showed a statistical difference in spectrum scores between groups (Kruskal-Wallace test *P* < .0001) (Fig. 1). On this day, the bacteria culture-negative group had a higher spectrum score than the *Enterobacterales* and *Stapylococcus aureus* groups (Dunn post-hoc test *P* < .001). None of the other analyzed days showed statistically significant differences in spectrum scores between groups (Kruskall-Wallace test *P* > .10) (Fig. 1). A significantly higher percentage of patients with positive bacterial cultures (70.3%) than bacteria-culture–negative patients (56.1%) remained on antibiotics at day 7 (χ^2^ test *P* < .001).

The pathogen groups exhibited outcomes that were not significantly different based on a composite of all-cause mortality and hospital readmission within 30 days of pneumonia diagnosis (χ^2^ test *P* = .049; χ^2^ post-hoc test *P* > .10). Moreover, 26.1% of patients in the *Staphylococcus aureus* group, 34.8% in the bacteria-culture–negative group, 40.2% in the *Enterobacterales* group, and 44.1% in the *Pseudomonas aeruginosa* group reached the composite end point.

## Discussion

The result of this retrospective cohort study provide no evidence that antibiotic de-escalation occurs at different rates based on the pathogen identified to be causative of HAP or VAP. This information is important because differences in treatment patterns between pathogen groups would have merited further study. Along with previous studies indicating that patient outcomes are no worse when antibiotic therapy is de-escalated, our data indicate that opportunities for further de-escalation can be pursued based on microbiologic pathogen type.^4–6^ Most interestingly, bacteria-culture–negative patients did not have lower rates of de-escalation compared to patients with an identified bacterial pathogen.

Our study had several limitations. The study included the small number of organisms recovered, predisposing to a type 2 error in de-escalation rates between groups and the inherent difference in antibiotic spectrum based on positive cultures driving de-escalation. That is, stopping linezolid versus meropenem when MRSA or ESBL gram-negative organisms, respectively, were not identified. Additionally, it is possible more patients in the culture negative group did not have pneumonia. This diagnostic uncertainty may have contributed to continued empiric antibiotic therapy, impeding any attempt at de-escalation. Finally, there are potential limitations of evaluating antibiotic spectrum rather than a broader off-target impact of antibiotics on microbiota which may more directly influence antibiotic resistance emergence and outcome.^8^

Antibiotic stewardship remains a challenging issue in the present time, especially among patients facing life-threatening conditions or those in ICUs. A recent cluster-randomized trial from a large US academic center found that weekly multidisciplinary antimicrobial stewardship round making was a high-resource intervention associated with a small impact on antibiotic utilization.^9^ Another recent study from Singapore found that an intensive antimicrobial stewardship program employing prospective review and computerized decision support systems was associated with limiting the use of pipercillin-tazobactam and carbapenems while reducing use of other antibiotics as well.^10^ Our findings suggest opportunities for further de-escalation that can occur based on quantitative assessments of antibiotic spectrum, especially for patients with bacteria-culture–negative HAP and VAP.
